# Differential privacy EV charging data release based on variable window

**DOI:** 10.7717/peerj-cs.481

**Published:** 2021-04-22

**Authors:** Rixuan Qiu, Xiong Liu, Rong Huang, Fuyong Zheng, Liang Liang, Yuancheng Li

**Affiliations:** 1 State Grid Jiangxi Information & Telecommunication Company, Nanchang, Jiangxi, China; 2 School of Control and Computer Engineering, North China Electric Power University, Beijing, Beijing, China

**Keywords:** Differential privacy, Dynamic release, V2G network, Privacy protection

## Abstract

In the V2G network, the release and sharing of real-time data are of great value for data mining. However, publishing these data directly to service providers may reveal the privacy of users. Therefore, it is necessary that the data release model with a privacy protection mechanism protects user privacy in the case of data utility. In this paper, we propose a privacy protection mechanism based on differential privacy to protect the release of data in V2G networks. To improve the utility of the data, we define a variable sliding window, which can dynamically and adaptively adjust the size according to the data. Besides, to allocate the privacy budget reasonably in the variable window, we consider the sampling interval and the proportion of the window. Through experimental analysis on real data sets, and comparison with two representative w event privacy protection methods, we prove that the method in this paper is superior to the existing schemes and improves the utility of the data.

## Introduction

With the rapid development of new energy technologies, electric vehicles have gradually become popular. Due to the limitation of on-board battery technology, EVs need to frequently visit charging stations for charging. During the interaction between the EV and the charging pile, a large amount of time data generated by the user is collected (battery status, payment records, the real identity of the electric vehicle user, the current location of the electric vehicle (Wang et al., 2019, etc.). These data can be released to third parties to make valuable decisions (charging service providers provide users with personalized services, electric vehicle load forecasts to guide grid dispatching work, etc.). But big data may be related to user’s sensitive information, including lifestyle, habits, and personal preferences, and third-party access to this information may cause privacy issues (Langer et al., 2013).

A common scenario for privacy protection of data release is that a trusted server collects a large amount of data from the client and continuously shares the aggregated data with other untrusted entities for various purposes. The data publisher must ensure that personal privacy is not leaked, so the corresponding privacy protection mechanism needs to be applied to the data before the data is released. The issue of private data release has attracted the attention of researchers across multiple disciplines. At present, the main method is to protect data release through differential privacy. Differential privacy (Dwork et al., 2006) requires the output of data query results to be roughly the same, so that the opponent cannot judge whether a single tuple in the database has been modified. As far as we know, the differential privacy protection of data stream publishing has gradually been applied to various streaming scenarios (Zhu et al., 2017), such as disease monitoring, traffic monitoring and social networking, but there are relatively few related studies in the smart grid.

In response to the related problems and application research needs in dynamic data publishing, we propose a new privacy protection mechanism with differential privacy data stream publishing. Specifically, our method is divided into four modules, using data prediction to adaptively sample the data stream, and then we design a variable window according to the data change rate, taking into account the impact of the variable window and the impact of the next window data dynamic change proposes a window-based dynamic budget allocation algorithm. Then, in order to reduce the interference error, the sampling points are clustered and grouped at each time stamp, and finally Kalman filter is used to reduce the publishing error.

The rest of the paper is organized as follows. In the second section we introduce the work related to data release, in the third section we introduce the basic knowledge of the system model and differential privacy, the fourth section introduces the module design and specific algorithms of our scheme, and the fifth section introduces our experiment and evaluation results. The seventh section summarizes the results.

## Related Work

In this section, we briefly review the latest research on data release based on differential privacy.

Some existing differential privacy methods focus on the release of static data (Liu, Shen & Sang, 2015; Jayabalan & Rana, 2018; Lu, 2016) for multi-sensitive attribute data, the idea of clustering and grouping is used to anonymize the data to protect sensitive data. To further improve the query accuracy (Kang et al., 2016) proposed a differential privacy publishing method based on an interval tree structure and adopted heteroscedasticity plus noise strategy for the data. However, these methods are all aimed at static data publishing. They cannot solve the problem that the privacy budget is easily exhausted under dynamic data streams, so they cannot be directly applied to data streams. Literature (Han & Xiao, 2016) proposes that the protection of charging and discharging privacy is an important task that needs to be studied in the future. At the same time, how to ensure the online disclosure of the charging and discharging data of electric vehicle users and the online disclosure of electronic bills is also a problem to be solved. However, no specific solutions and methods were given. Literature (Kellaris et al., 2014) combines event-level differential privacy and user-level differential privacy and proposes a new concept of w-event privacy on an infinite stream. W-event privacy uses a sliding window mechanism to protect w consecutive events that occur in a sliding window. The privacy of any sequence of events at a point in time. Literature (Cao et al., 2017) quantified the differential privacy risk under time correlation for the influence of time correlation in a continuous data release (Acs & Castelluccia, 2014; Ma et al., 2019; Wang, Sinnott & Nepal, 2017; Jo, Jung & Park, 2018). studied the release of user trajectory data, and proposed a space-based differential privacy protection mechanism through the spatial correlation of trajectory data, but it is not applicable. Literature (Yong et al., 2018) defines a new concept to measure the utility of published data and proposes an adaptive w-event differential privacy model based on this, but it does not consider non-sampling point errors (Chen et al., 2017). Proposed PeGaSus, which uses the perturbation group smoother framework to smooth excessive noise, thereby improving the utility of the data (Wang & Li, 2019). To reduce the consumption of privacy budget, the wireless data stream is released through a simple random sampling Laplace mechanism, but when the stream data is too long, it cannot maintain good utility (Fan & Xiong, 2014) for the high correlation between time series data it may lead to the problem of the low utility of data released by differential privacy. The Kalman filter is used to filter and adaptively sample data, but it cannot protect the privacy of unlimited data streams (Ren, Zhang & Zhang, 2019). The controller design method is studied, through the controller design to adjust the shape of the system probability density function (Tang, Zhang & Hu, 2020). Through PID and extended Kalman filter to dynamically adjust the set point, the performance enhancement scheme of the nonlinear system is proposed to meet the control design requirements under random noise (Wang et al., 2019). Proposed an improved Kalman filter differential privacy stream data release scheme, which can adjust and calibrate the prior estimates caused by sudden changes in data. Literature (Li, 2015) proposes an adaptive sampling method based on distance, which solves the problem of large accumulated errors in real-time publishing of differential privacy dynamic data sets. Literature (Wang et al., 2018) proposed a data release scheme with w event privacy protection (RescueDP). Its structure includes adaptive sampling, adaptive budget allocation, dynamic grouping, disturbance and filtering, and seamless integration as a whole. To sum up, the research on the privacy protection of dynamic data release has some methods and measures, but there are few gaps in the privacy protection model for the dynamic data that needs to be disclosed and released in the V2G network.

## Preliminaries and Problem Statement

### Differential privacy

**Definition 1** Differential privacy. Given adjacent data sets D and D′, they differ by at most one record from each other. Given a privacy algorithm A, O is the value range of A. If algorithm A outputs the result range S on data sets D and D′ arbitrarily, where S belongs to O and satisfies the following inequality, then algorithm A satisfies *ϵ*-differential privacy, and its privacy can be measured by the parameter *ϵ*.

(1) \begin{eqnarray*}\Pr\nolimits \left[ \mathrm{A} \left( \mathrm{D} \right) =\mathrm{O} \right] \leq \exp \nolimits (\epsilon )\times \Pr\nolimits [\mathrm{A} \left( {\mathrm{D}}^{{^{\prime}}} \right) =\mathrm{O}]\end{eqnarray*}

**Definition 2** Global Sensitivity. For adjacent data sets D and D′, for any query function *f*:*D* → *R*^*d*^, the global sensitivity of the query function is:

(2) \begin{eqnarray*}\mathrm{GS}=\max \nolimits (\mathrm{D},{\mathrm{D}}^{{^{\prime}}}){ \left\| f \left( \mathrm{D} \right) -f({\mathrm{D}}^{{^{\prime}}}) \right\| }_{1}\end{eqnarray*}

Among them, *R* represents the real number space mapped by the function, and *d* represents the query dimension of the function *f*.

The noise mechanism is the main technology to achieve differential privacy protection, and the commonly used noise addition mechanism is the Laplace mechanism. The noise level required by the algorithm based on this mechanism and satisfying differential privacy is closely related to the global sensitivity.

**Theorem 1** Laplace mechanism. For any functions *f*:*D* → *R*^*d*^, if the output result of algorithm A satisfies the following equation, then A satisfies *ϵ*-differential privacy:

(3) \begin{eqnarray*}\hat {f} \left( D \right) =f \left( D \right) +Lap( \frac{GS}{\epsilon } )\end{eqnarray*}

Among them, $Lap( \frac{GS}{\epsilon } )$ represents the added Laplace noise.

### w-Event Privacy

**Definition 3** Let A be a random algorithm and Y as all possible outputs of algorithm A. For all w-neighbors’ data streams *S*_*t*_ and ${S}_{t}^{\mathrm{` }}$, if it satisfies:

(4) \begin{eqnarray*}\Pr\nolimits \left[ \mathrm{A} \left( {S}_{t} \right) \in \mathrm{Y } \right] \leq \exp \nolimits (\epsilon )\times \Pr\nolimits [\mathrm{A} \left( {S}_{t}^{\mathrm{` }} \right) \in \mathrm{Y }]\end{eqnarray*}

Then algorithm A satisfies w-event privacy. To meet the above conditions, the privacy budget ε of algorithm A should satisfy the condition of ${\mathop{\sum }\nolimits }_{t-w+1}^{t}{\epsilon }_{i}\leq \epsilon $ within w consecutive timestamps. This theory makes w-event privacy considers ε as the total privacy budget in a window of any size.

### Problem statement

**Figure 1:**
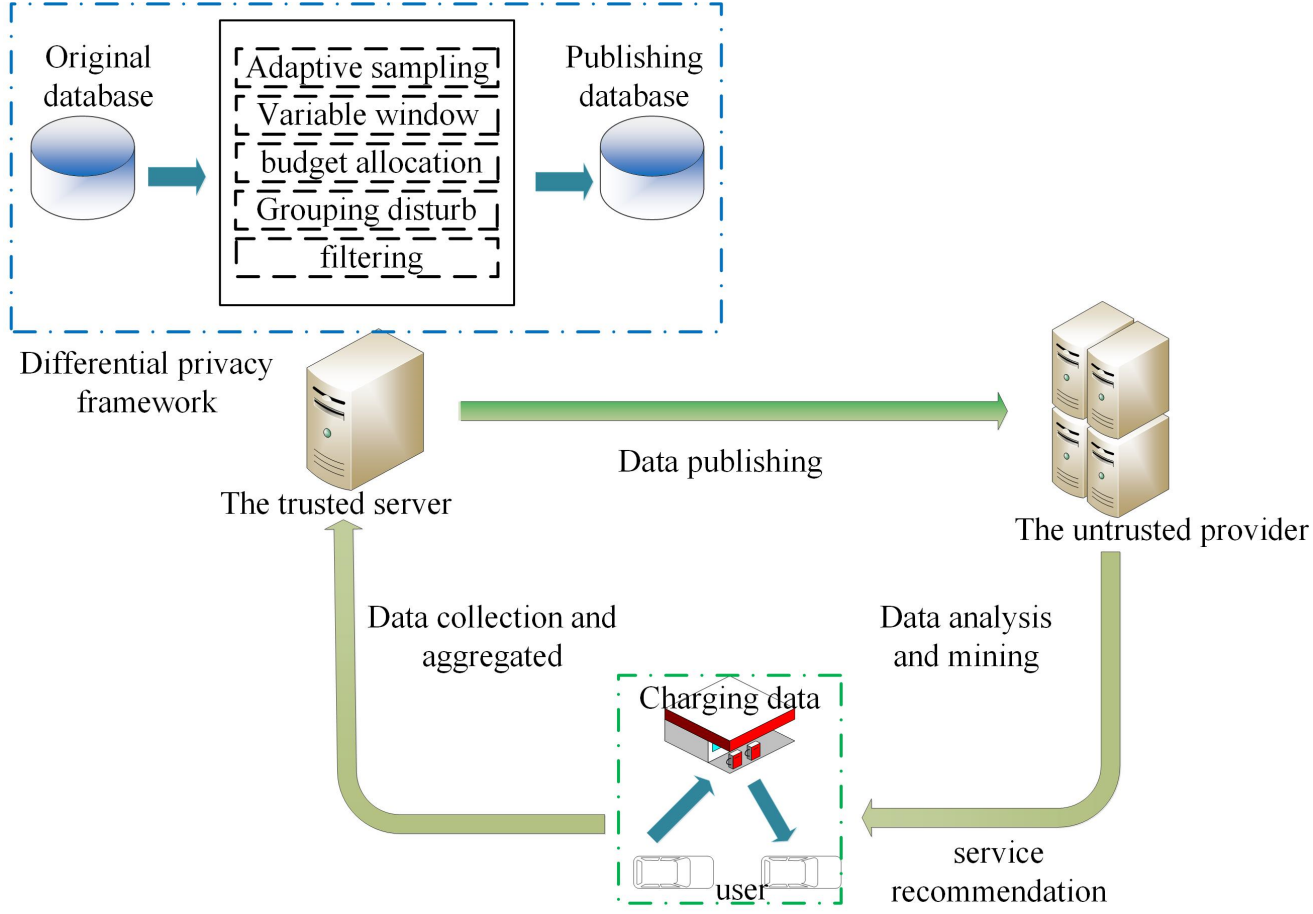
System model.

In the data release model in the V2G network in Fig. 1, BVs charge at the corresponding charging station to generate information interaction, which contains user privacy information (account ID, charging data, etc.), and then the aggregator collects the corresponding data information from the charging station to aggregate and periodically report the aggregation results to the central server. The central server establishes a real-time database based on these database files, and the data are collected and stored by the trusted server. The goal is to continuously publish the charging data on the database under the condition of a w-event privacy guarantee. Therefore, the trusted server does not release the original value of the charging data but applies a differential privacy mechanism to release the purified version of the original charging data to a third party for data analysis and mining.

We define the original data stream D as an infinite sequence of tuples. The definition of each tuple is (s, n, t), and all tuples form a domain dom = *S* × *N* × *T*, Here *S* represents the station of the charging station, *N* represents the set of charging capacity, and *T* is the infinite time stamp. Each tuple (s, n, t) represents the charging capacity of a certain charging station in the t time stamp, and each data stream may be the charging capacity of different users at different time stamps. It is worth noting that t does not describe a specific instantaneous event, but an aggregation behavior of a time step. Since users do not want to disclose their information, a clean version of the original data is released for privacy protection.

### Mechanism architecture

**Figure 2:**
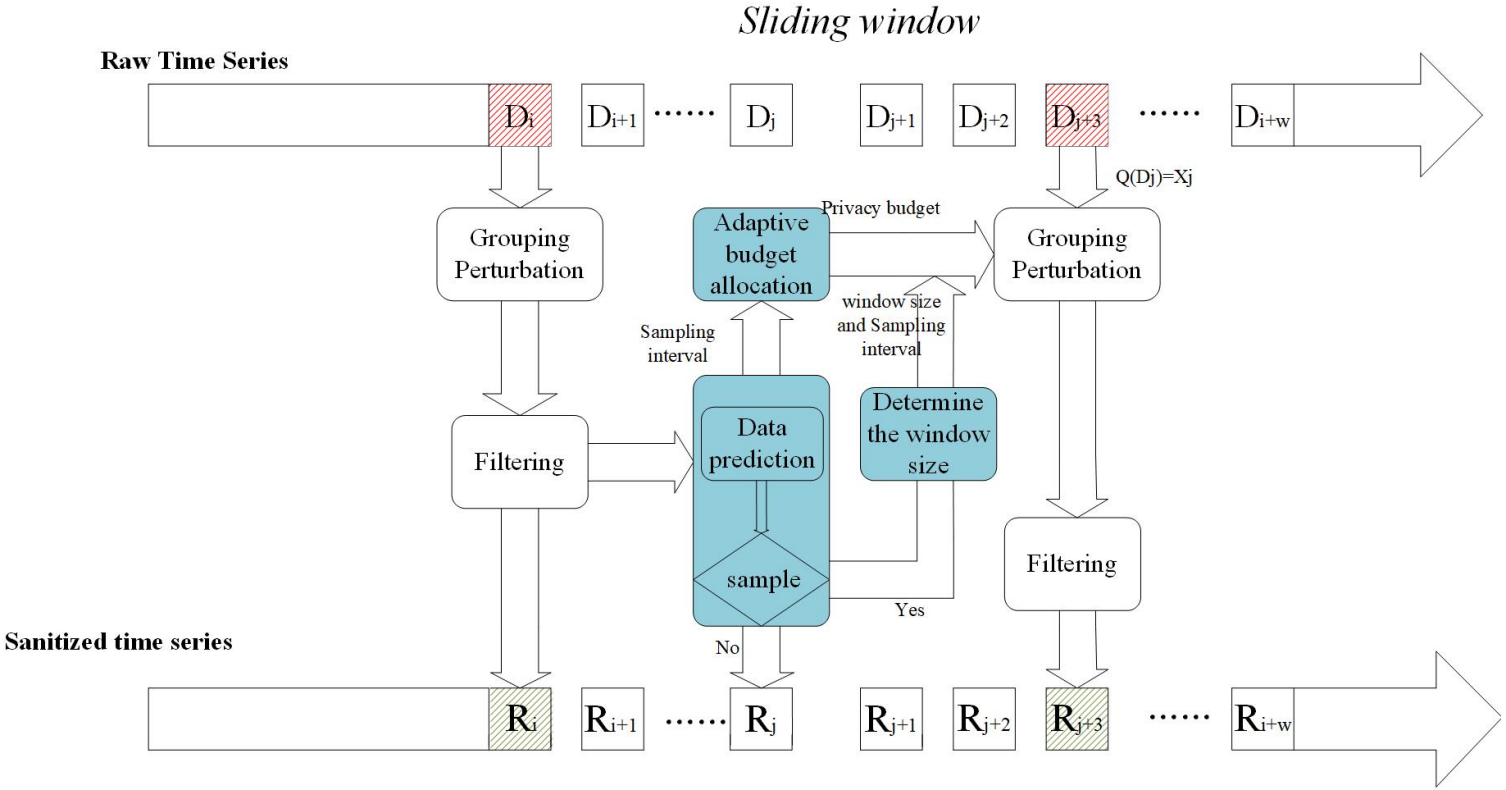
Mechanism architecture.

In this section, we propose a real-time charging data publishing algorithm based on w-event differential privacy. Figure 2 shows the framework of the proposed scheme, which contains five modules: packet perturbation, adaptive sampling, variable sliding window, adaptive budget allocation, and filtering.

First, the adaptive sampling mechanism adjusts the sampling frequency according to the data dynamics and data prediction model to determine the sampling points and then judges the data dynamics according to the sampling frequency to determine the size of the window. The next step is to allocate a privacy budget to the sampling points according to the window size and the sampling interval to ensure differential privacy. For non-sampling points, approximate by publishing their predicted values. Then the sampling points are clustered and grouped to add noise. Finally, the introduction of the Kalman filter filtering mechanism is beneficial to improve the overall accuracy.

#### a. Adaptive sampling

For data stream differential privacy release, every release of noisy data requires a certain privacy budget. The data stream itself has the characteristics of continuity, and if noise is added to each timestamp, it will inevitably cause large error. To minimize the disturbance error, the general strategy is to sample the data stream. In this paper, the data dynamics are detected by the predicted value of the data prediction model, and the sampling interval is reduced when the data changes rapidly, and vice versa. We use the ARIMA prediction model (Wu, Yang & Chen (2019)) to predict the data. First, the charging time series are differentiated to obtain the stationary series *X*_*t*_. According to AIC and BIC, we find the optimal model (the AIC and BIC function values reach the minimum) to determine the model order, and perform a white noise test on the model residual sequence that has been created. If the test fails, reselect the model to fit, and finally check the future Time series for forecasting.

The ARIMA model is generally denoted as ARIMA(p,q,d), which represents the stationary sequence *X*_*t*_ after d difference processing is performed on the charging data sequence *Y*_*t*_. After that, the stationary series *X*_*t*_ is fitted into an ARMA(p,q) model, which is expressed as follows:

(5) \begin{eqnarray*}{\hat {y}}_{t}={\phi }_{1}\ast {X}_{t-1}+\ldots +{\phi }_{p}\ast {X}_{t-p}+u-{\theta }_{1}\ast {e}_{t-1}-\ldots -{\theta }_{q}\ast {e}_{t-q}\end{eqnarray*}

In the formula: the first half is the autoregressive process; p is the autoregressive order; *ϕ* is the autoregressive coefficient; The second half is the moving average process; q is the moving average order; *θ* is the moving average coefficient; *X*_*t*_ is a stationary data series; *e*_*t*_ is the unobserved white noise sequence ${e}_{t}={y}_{t}-{\hat {y}}_{t}$, *X*_*t*_ is the d-order difference of the time series data *y*_*t*_.

In particular, when *q* = 0, the model becomes the autoregressive model AR (p), and the expression is as follows:

(6) \begin{eqnarray*}{X}_{t}={\phi }_{1}\ast {X}_{t-1}+\ldots +{\phi }_{p}\ast {X}_{t-p}+{e}_{t}\end{eqnarray*}

When *p* = 0, the model becomes a moving average model MA (q), the expression is as follows:

(7) \begin{eqnarray*}{X}_{t}={e}_{t}-{\theta }_{1}\ast {e}_{t-1}-\ldots -{\theta }_{q}\ast {e}_{t-q}\end{eqnarray*}

The prediction model can obtain the predicted value ${\hat {y}}_{t}$ of the current timestamp through the historical data for the sampling algorithm.

**Table 1:** Algorithm 1.

Sampling algorithm
**Input:** The current privacy budget *ϵ*_*i*_, the predicted value ${\hat {y}}_{i}^{j}$, the release data at the last sampling timestamp ${\tilde {x}}_{l}^{j}$
**Output:** Sampling or not
Calculation $\mathrm{dis}= \left\vert {\hat {y}}_{i}^{j}-{\tilde {x}}_{l}^{j} \right\vert $
Calculation ${\lambda }_{i}^{j}=1/{\epsilon }_{i}$
**If**dis > *λ*_*i*_, then
Set i as the sampling point, update sampling interval
I =i −*l*
**Else**
i is not a sampling point, release predictive value ${\hat {y}}_{i}^{j}$
**End if**

As shown Algorithm1 in the Table 1, for any timestamp *t*, first we obtain its predicted data through the prediction algorithm, and secondly calculate whether t is a sampling timestamp based on the difference from the previous publishing node. If it is not the sampling point, directly publish the forecast data. If it is a sampling point, a privacy budget is allocated according to differential privacy requirements.

#### b. Variable window

In the existing dynamic data publishing based on sliding windows, algorithms mostly use fixed-length sliding windows. However, the data stream is always fluctuating, with fluctuating periods and smooth periods. For example, within consecutive timestamps , the charging data of a public charging station changes greatly during the day, but it is relatively stable at night. To reflect this data change and reduce the release error, we propose a variable-size sliding window, which can adjust the window size adaptively according to the dynamic changes of the data stream, which is more flexible. Intuitively speaking, when the data is flat, there are relatively few sampling points. We need to expand the sliding window to include more data. When the data changes rapidly, reduce the window size.

Specifically, we predefine the optimal sampling frequency within a period, and then calculate and inspect the sampling frequency within a period (the sampling points are obtained according to the sampling model), and compare the difference with the optimal sampling rate to reflect the current period data Rate of change. We use PID (Proportional-Integral-Derivative), a commonly used control loop feedback mechanism to dynamically adjust the window size. We define the PID error calculation formula as follows:

(8) \begin{eqnarray*}{\delta }_{i}={C}_{p}\times {E}_{i}+{C}_{i}\times \sum _{o=n-\pi -1}^{n}{E}_{o}+{C}_{d}\times \frac{{E}_{i}-{E}_{j}}{{t}_{i}-{t}_{j}} {C}_{p}+{C}_{i}+{C}_{d}=1,{C}_{p},{C}_{i},{C}_{d}\geq 0\end{eqnarray*}

The parameter ${E}_{i}= \left\vert \frac{{n}_{i}}{M} - \frac{n}{N} \right\vert $ is the feedback error, $ \frac{n}{N} $ is the defined optimal sampling frequency, *M* is the number of timestamps in the current period, and *n*_*i*_ is the number of sampling points in the *M* timestamp. For the convenience of calculation, we calculate *M* according to the number of sampling points to obtain the current sampling frequency. The first term of PID error *C*_*p*_ × *E*_*i*_ is the proportional term representing the proportional error of the current error, the second term ${\mathop{\sum }\nolimits }_{o=n-\pi -1}^{n}{E}_{o}$ is the integral term representing the integral error of the past accumulated error, *E*_*o*_ is in The error at the sampling point *t*_*o*_, the third term ${\theta }_{d}\times \frac{{E}_{i}-{E}_{j}}{{t}_{i}-{t}_{j}} $ is the derivative error of the derivative term predicting the future error.

From the above formula, we can find the PID error, which feedbacks the size of the sliding window according to the data change rate. However, when the data changes slowly, the sliding window with fewer sampling points will contain more data items, especially non-sampling points. Non-sampling points are published with their predicted values, and the error will gradually increase as the window increases, so we introduce non-sampling point errors to constrain the window size:

(9) \begin{eqnarray*}{W}_{t}=\mathrm{N}+\text{sign} \left( \frac{n}{N} - \frac{{n}_{i}}{M} \right) \times \theta \times {\delta }_{i}-\beta \sum _{\gamma =i-s+1}^{i}{e}_{\gamma }\end{eqnarray*}

Here sign(.) is a sign function, which controls the window is expand or shrink. The parameters *θ* and *β* respectively determine the influence of PID error and cumulative error of non-sampling points. *e*_*γ*_ represents the prediction error, where the parameter $s=\mathrm{N}+\text{sign} \left( \frac{C}{N} - \frac{{C}_{i}}{M} \right) \times \theta \times {\delta }_{i}$, note that *e*_*γ*_ here does not include sampling points.

Due to the variability of the window, when we slide the window between two adjacent windows, the sliding step of the left end of the window is the interval between sampling points and the right end of the window controls the size of the window.

#### c. Privacy budget allocation

W-event privacy requires that the sum of the budget in any window with a step size of w does not exceed the entire privacy budget *ϵ*. The existing privacy budget allocation mechanism does not apply to the method we proposed. For example, the BA and BD mechanisms rely on an assumption that the data will not change significantly within consecutive timestamps, which is not in line with the real scene. RescueDP uses dynamic data changes to allocate privacy budgets. Consider our method. When the data changes from fast to slow, our adaptive window size changes from small to large, but the remaining budget at the sampling point where the data changes slowly are small. Introduce larger errors and vice versa.

The aforementioned existing privacy budget allocation algorithms all assume a fixed window size and are not suitable for adaptive size window technology. In this article, as with the previous technology, we consider the dynamic changes of data to allocate the privacy budget (Wang et al., 2018), but we have to consider the adaptive change of the window on this basis. Specifically, we calculated the weight ratio of the sampling interval of the current sampling point in the window. If the sampling interval of the sampling point at the current moment occupies a larger ratio of the window, we judge that the data has a tendency to change slowly, so we assign it to the current node A relatively small privacy budget is used to reduce the error due to the remaining more privacy budget at the later sampling points. Vice versa.

It can be seen from Algorithm 2 in the Table 2 that for a sampling point, it is firstly necessary to determine whether it is within the range of the window because the length of the window at each moment is not the same. If the sampling point is not in the window, then we need to slide the window (remove a sampling point from the left end of the window) to update the length of the window until the window contains the current sampling point and allocate a privacy budget. It can be seen from the privacy budget allocation formula that we use the natural logarithmic coefficient ln(*I* + 1) to describe the relationship between the sampling interval and the privacy budget. To consider the data changes in the next window, the coefficient $(1- \frac{I}{w} )$ the value range is between (0, 1). When *I* becomes larger, it becomes smaller to constrain the remaining privacy budget and vice versa.

**Table 2:** Algorithm 2.

Privacy budget allocation
**Input:** sampling interval I, Privacy budget *ϵ*, Privacy budget allocated for each sampling point $ \left( {\epsilon }_{1},\ldots ,{\epsilon }_{i-1} \right) $
**Output:** Privacy budget *ϵ*_*i*_ for sampling point *i*
**if***t*_*i*_ not included in the window *W*_*t*_
Calculate the number of sampling points *n*_*i*_
Calculate the current sampling frequency $ \frac{{n}_{i}}{M} $ and update *W*_*t*_
Calculate the remaining privacy budget ${\epsilon }_{\mathbi{r}}=\epsilon -{\mathop{\sum }\nolimits }_{\mathbi{k}=\mathbi{i}-\mathbi{w}+\mathbf{1}}^{\mathbi{i}-\mathbf{1}}{\epsilon }_{\mathbi{k}}$
Calculate the allocated privacy budget ${\epsilon }_{\mathbi{i}}={\epsilon }_{\mathbi{r}}\times (\mathbf{1}- \frac{\mathbi{I}}{\mathbi{w}} )\cdot \mathbf{ln}(\mathbi{I}+\mathbf{1})$

#### d. Grouping Perturbation

Inspired by RescueDP, instead of directly adding Laplace noise to the sampling point when perturbing each sampling point, we group each time stamp *t*_*i*_ data and add Laplace noise to the grouping result. In this paper, the FCM clustering algorithm (Zheng et al., 2020) is used to group all the sampling points at this moment (non-sampling points do not need to add noise and do not group). To protect data privacy, the algorithm uses the predicted values of the sampling points instead of the original data values.

In Algorithm 3 in the Table 3, the threshold *τ* reflects whether the value has sufficient anti-noise ability. Specifically, the input is the predicted value of the site at each timestamp *t*_*i*_. Firstly, the Euclidean distance between the data point and the cluster center is calculated according to the cluster center, and then the membership matrix of the data point is obtained. It represents each the degree to which a data point belongs to a certain cluster is in the interval (0, 1). Then the value function is calculated. If it is less than a certain threshold, or the amount of change from the previous value function value is less than a certain threshold, the algorithm stops, otherwise, a new membership matrix is calculated. To meet the differential privacy requirements, Laplace plus noise is performed on the minimum privacy budget of each group and then averaged to each value in the group.

**Table 3:** Algorithm 3.

Grouping perturbation algorithm
**Input:** The data set *S*_*t*_*i*__ that needs to be sampled at the time *t*_*i*_, the Predicted value of the sampling node ${\hat {x}}_{i}^{j}$
**Output:** The noise value of sampling point in *S*_*t*_*i*__
**for** each site j ∈ *S*_*t*_*i*__**do**
Predict statistics ${\hat {x}}_{i}^{j}$ using the ARIMA model
**If**${\hat {x}}_{i}^{j}\gt \tau $**then**
Let the site j itself as a group, add the group to *G*_*t*_*i*__
**Else**
add the site j into Φ
Initialize cluster centers *c*_*i*_
**while**J not convergent **do**
Initialize Membership matrix ${U}_{ij}= \frac{1}{{\mathop{\sum }\nolimits }_{k=1}^{c}{ \left( \frac{ \left\| {x}_{j}-{c}_{i} \right\| }{ \left\| {x}_{j}-{c}_{k} \right\| } \right) }^{ \left( \frac{2}{m-1} \right) }} $
Calculated value function $\mathrm{J}={\mathop{\sum }\nolimits }_{i}^{c}{\mathop{\sum }\nolimits }_{j=1}^{n}{U}_{ij}^{m}{ \left\| {x}_{j}-{c}_{i} \right\| }^{2}$
Revised cluster centers
Cluster result: $\mathrm{P}= \left\{ {\mathrm{C}}_{\mathrm{j}}:j \text{is a cluster center} \right\} $
Group the site in Φ according to P and add each group to *G*_*ki*_
Introduce Laplace noise into each group *G*_*t*_*i*__
Allocate the group perturbed statistic to each site according to ${\hat {x}}_{i}^{j}$
$A \left( g \right) ={\mathop{\sum }\nolimits }_{j=1}^{k}\sum g \left[ j \right] +Lap \left( \frac{\Delta \left( f \right) }{\min \left( {\epsilon }_{g \left[ j \right] } \right) } \right) $
**return** The noise value $A \left( g \left[ j \right] \right) =A \left( g \right) /k$ of sampling point in *S*_*t*_*i*__

#### e. Filtering

To reduce the errors caused by using published data in privacy budget allocation and grouping, we use Kalman filtering to improve the accuracy of data publishing. Kalman filtering has been proven in FAST to improve the accuracy of data stream release. In the final release result, it uses the state-space model to implement posterior estimation (prediction and correction) of the sampling points, and we omit the filtering details. Please refer to (Fan & Xiong, 2014) for detailed steps.

#### f. Privacy Analysis

**Theorem 2**Proposed satisfies w-event privacy.

Proof. In the method framework proposed in this paper, only privacy budget allocation and the grouping algorithm have an impact on data disturbance. Therefore, we only need to prove that these two modules satisfy w-event differential privacy, and our method is to satisfy w-event differential privacy. In the privacy budget allocation algorithm, we do not allocate privacy budgets to non-sampling points, while for sampling points we calculate the remaining privacy budget in the calculation window and then allocate it to the corresponding sampling points ${\epsilon }_{r}\times (1- \frac{I}{w} )\cdot \ln (I+1)$, the total privacy budget in each w step window ${\mathop{\sum }\nolimits }_{k=i-w+1}^{i-1}{\epsilon }_{k}\lt \epsilon $, because we always allocate part of the remaining privacy budget to the sampling points in the window. For the grouping algorithm, we divide the sampling points at the same time into n groups, and for each group, we add noise in the following way $A \left( {g}_{1} \right) ={\mathop{\sum }\nolimits }_{j=1}^{k}g \left[ j \right] +Lap \left( \frac{\Delta \left( f \right) }{\min \left( {\epsilon }_{g \left[ j \right] } \right) } \right) $, it can be known from theorem 1, $A \left( {g}_{1} \right) \text{satisfies}\min \left( {\epsilon }_{g \left[ j \right] } \right) $-differential privacy. According to Axiom 2.1.1 in (Kifer & Lin, 2010), as long as the post-processing algorithm does not directly use sensitive information, the data can be kept private after the post-processing purification. Therefore, we know that the privacy budget used for disturbance is not greater than the privacy budget allocated to the sampling points. So our method satisfies w-event differential privacy.

### Experimental and Analysis

#### Experimental setup

In this section, we compare the performance of our method with BA and RescueDP on real data sets. The BA method is a scheme with w-event privacy protection designed for real-time data publishing, while RescueDP is a classic strategy to provide w-event privacy for real-time aggregated data publishing. The experimental environment of all schemes is A10-7300 Radeon R6, 10 Compute Cores 4C+6G 1.90 GHz, 8GB RAM, Windows 10 Operating system, implemented in java. For the description and default values of the parameters used in the experiment, we set the PID parameters (*C*_*p*_, *C*_*i*_, *C*_*d*_) to (0.9,0.1,0), *π* to 5, At the beginning of the release, the privacy budget needs to be calculated based on the window size, so we set the initial value of the window *w* = 40. The minimum value of N is min(N)=40, and the grouping algorithm threshold *τ* is set to *τ*=40.

Datasets: Our data source comes from ACN-Data, which is a dynamic data set of EV charging sessions in the workplace. There are currently more than 80 EV charging ports in a garage on the Caltech campus, and the shared power limit is 300 kWh (enough to accommodate 42 conventional ports). The system currently charges 65 EVs per day on average, and in the past three years, it has delivered more than 2.3 million miles of charging. The data set contains information about each charging session from 3 different locations.

This article uses two evaluation standards to measure the availability of algorithms, namely the average relative error and the average absolute error. The average relative error and average absolute error are defined as follows:

(10) \begin{eqnarray*}\mathrm{MAE}& =\sum _{r\in R} \frac{ \left\vert \mathrm{D} \left( r \right) -{\mathrm{D}}^{{^{\prime}}}(r) \right\vert }{ \left\vert R \right\vert } \end{eqnarray*}

(11) \begin{eqnarray*}\mathrm{MRE}& =\sum _{r\in R} \frac{ \left\vert \mathrm{D} \left( r \right) -{\mathrm{D}}^{{^{\prime}}}(r) \right\vert }{ \left\vert R \right\vert \ast \mathrm{D} \left( r \right) } \end{eqnarray*}

Among them, $\mathrm{D} \left( r \right) $ is the original data stream, D′(*r*) is the data stream after interference, and *R* is the set of all timestamps.

### Result analysis

Figsures 3 and 4 show the average relative error and absolute error under different privacy budgets ε.In our experiment, we set the size of the sliding window of the two reference algorithms unchanged to *w* = 100 to compare the errors under different privacy budgets ε. As shown in the figure below, the value of the privacy budget ε ranges from 0.1 to 1.25. From the results, we can see that as the privacy budget increases, the average error and absolute error gradually become smaller. This is because as the privacy budget increases, the amount of noise added according to the Laplace mechanism decreases, and the corresponding error decreases, and the availability increases. Also, the method in this paper is better than BA, RescueDP, and MAE and MRE are much smaller.

**Figure 3:**
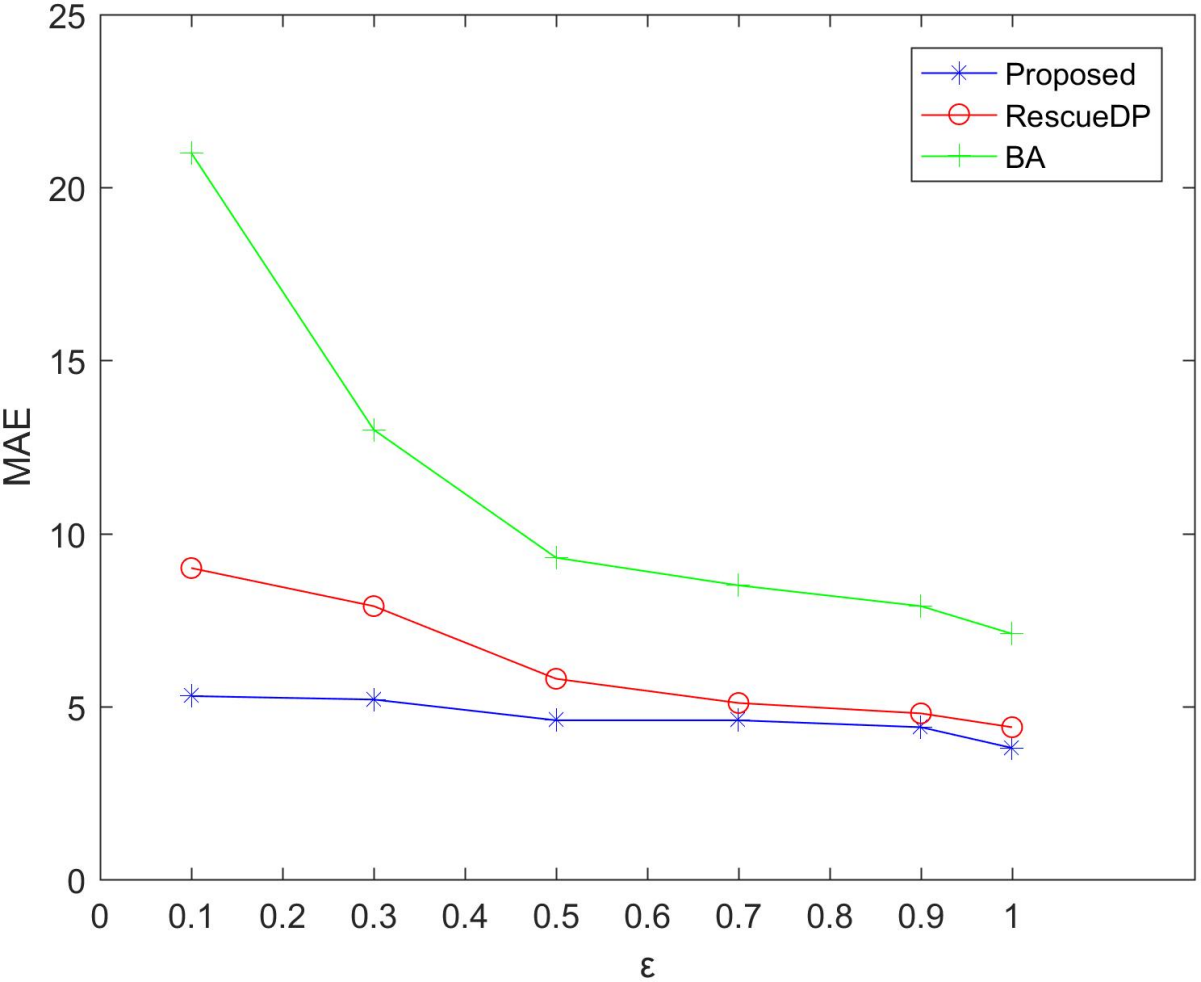
MAE under different privacy budgets.

**Figure 4:**
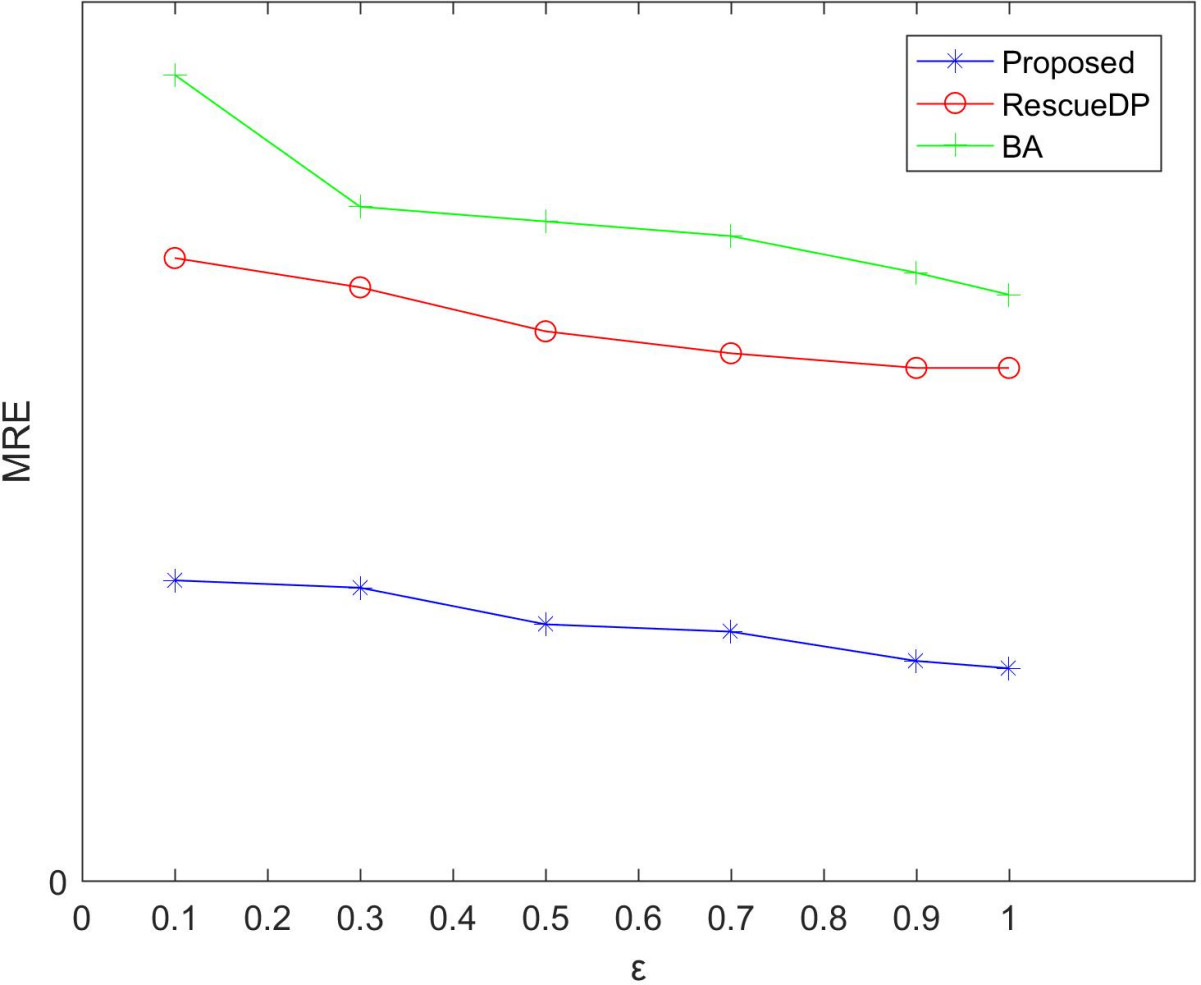
MRE under different privacy budgets.

The method in this paper is superior to other methods due to the following aspects. This is firstly due to the improved data availability by the adaptive window mechanism, and secondly, we consider the weight of the sampling interval in the corresponding window and have a rich privacy budget for each sampling point in the window. Also, cluster-based grouping algorithms provide better grouping results.

**Figure 5:**
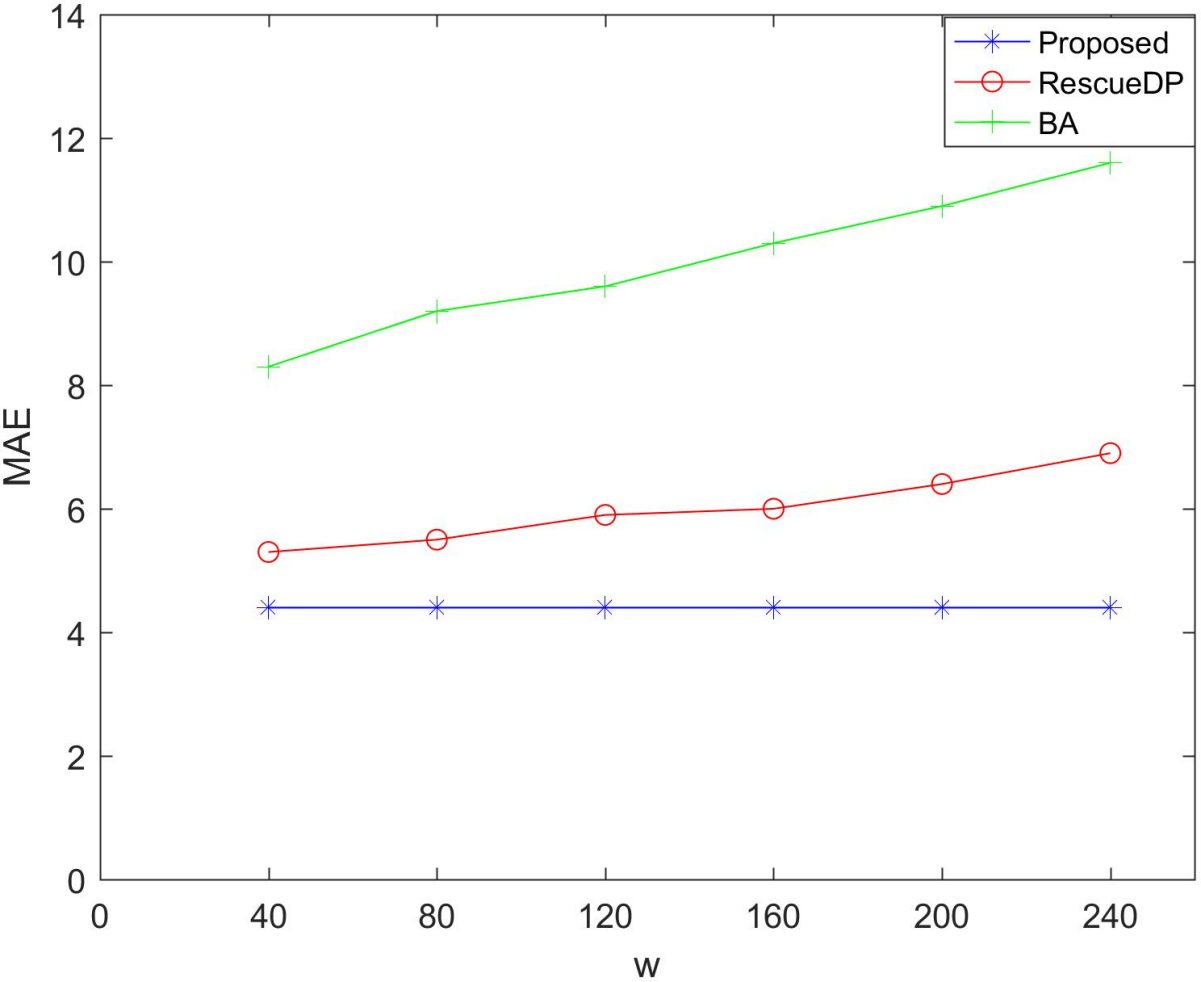
MAE under different windows size.

**Figure 6:**
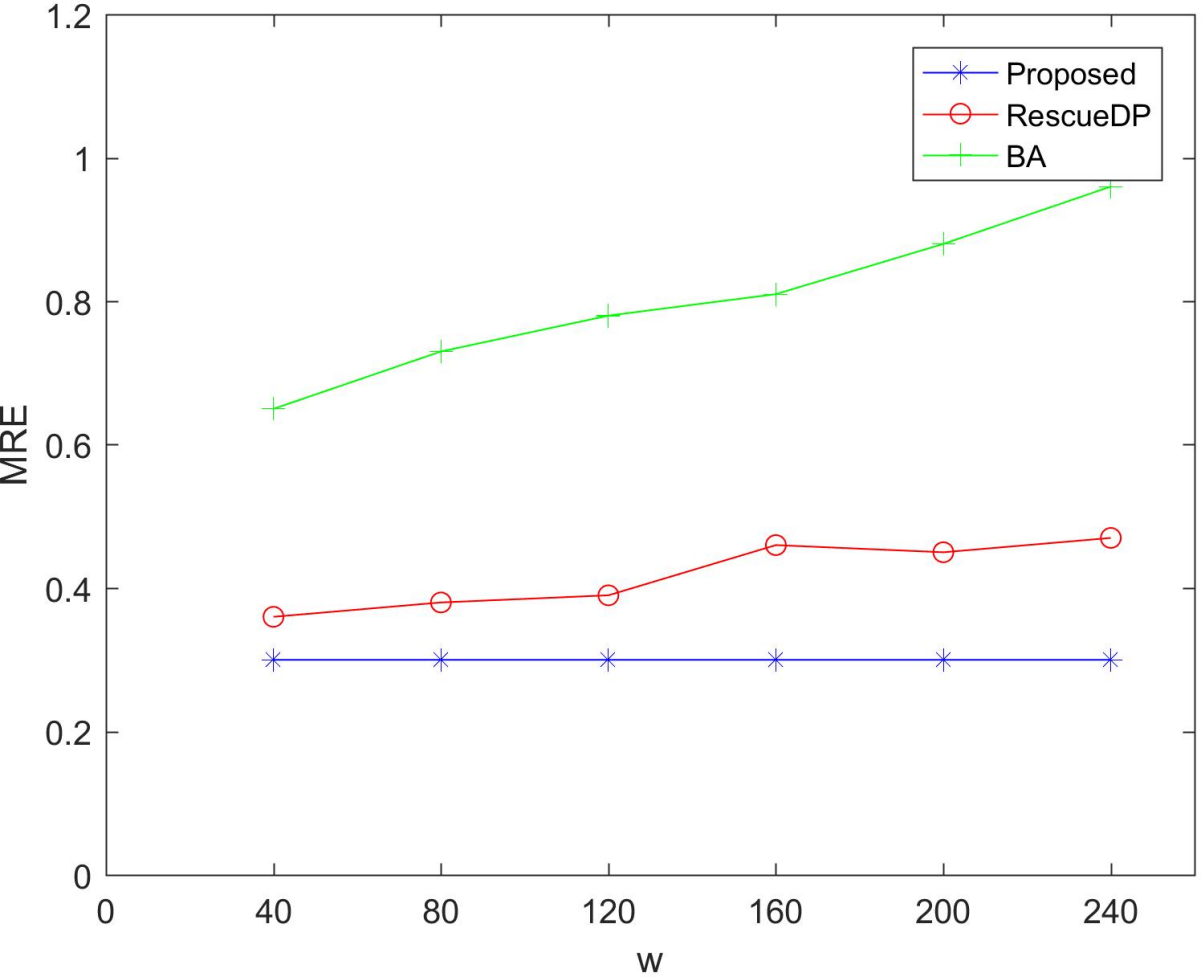
MRE under different windows size.

Figures 5 and 6 show the utility of adaptive windows. We set the privacy budget unchanged ε = 1, Comparing BA and RescueDP of different sliding window sizes, the range of the sliding window is 40 to 240. According to the results, it can be seen that as the window becomes larger, the error of BA and RescueDP will increase. The adaptive change of the window of the method in this paper will not change with the change of w. The utility will not be affected. The error curve approaches a horizontal straight line. This is because changing the window size increases the number of samples, the release error of BA and the approximate error of RescueDP increase exponentially, especially the absolute error increases significantly. It can be seen from the figure that the adaptive window error is smaller than the other two methods.

**Figure 7:**
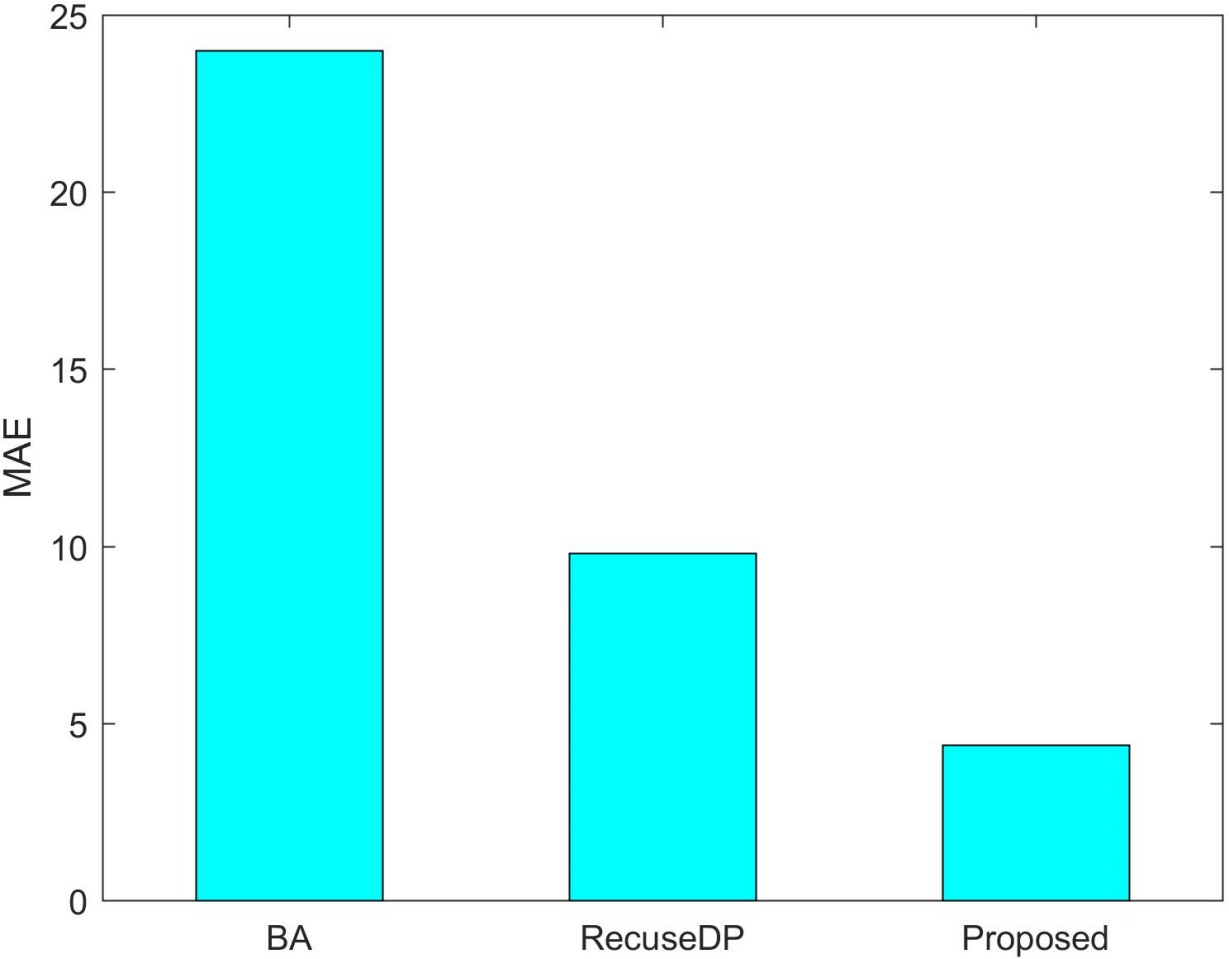
MAE under different budget allocation.

**Figure 8:**
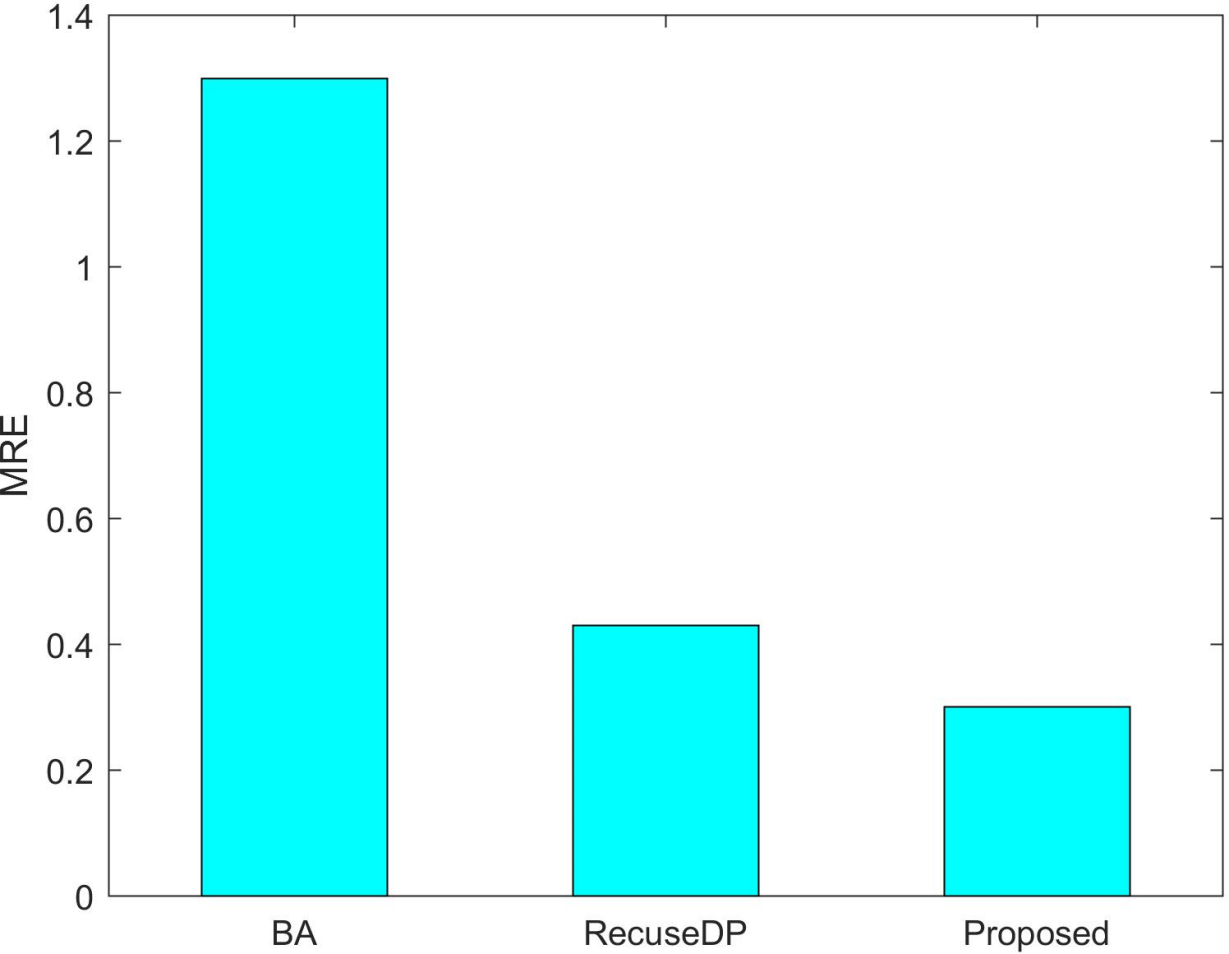
MRE under different budget allocation.

Figures 7 and 8 show the utility of the privacy budget algorithm. To evaluate the effect of the budget allocation algorithm, we use BA and RescueDP privacy budget allocation algorithms to allocate the privacy budget in the adaptive window, and the total privacy budget ε = 1 runs on our data set. It can be seen from the figure that the BA error is the largest. As the data increases, the allocable privacy budget decreases, which does not apply to the real situation. Then the error of RescueDP is larger than that of the method in this paper, because its privacy budget allocation does not consider the change of window size, and it is not suitable for the adaptive window. The method in this paper takes into account the sampling interval and window size, thereby avoiding errors caused by unreasonable privacy budget allocation.

Running time. Table 4 shows the running time of these methods in our data set. From the table, it can be seen that the running time of BA has accelerated thanks to its simple sampling and budget allocation mechanism, and our mechanism is these three methods the slowest one is because we need to readjust the window every time we slide the window. To reduce the error, we increase the computational cost.

**Table 4:** Comparison of running time.

	**BA**	**RecuseDP**	**Proposed**
Time	0.089s	0.171s	0.215s

## Conclusions

In this article, we propose a V2G network charging data release framework based on differential privacy to protect user privacy during the V2G network data release. Our framework includes adaptive sampling, variable windows, privacy budget allocation, packet perturbation, and filtering mechanisms. It can adaptively adjust the sampling frequency and dynamically adjust the window size according to the frequency of data changes. The privacy budget allocation scheme can allocate the privacy budget in a window of any size without quickly exhausting the privacy budget, and solves the problem that the existing privacy budget allocation does not apply to the variable sliding window. We further reduce interference errors and publishing errors through clustering and filtering mechanisms. We prove through theoretical analysis that the proposed scheme satisfies w-event privacy. Experiments on real data sets show that our method improves the effectiveness of time series publishing. Future work directions may include publishing data under untrusted servers, distributed privacy protection frameworks, and studying their privacy-utility trade-offs.

##  Supplemental Information

10.7717/peerj-cs.481/supp-1 Supplemental Information 1 Charging data

10.7717/peerj-cs.481/supp-2 Supplemental Information 2 Code

## Additional Information and Declarations

### Data Availability

The following information was supplied regarding data availability: Data is available at ACN-Data: https://ev.caltech.edu/dataset. The data and code are available as Supplementary Files.
